# Social Cognition and Interpersonal Problems in Persistent Depressive Disorder vs. Episodic Depression: The Role of Childhood Maltreatment

**DOI:** 10.3389/fpsyt.2020.608795

**Published:** 2021-01-25

**Authors:** Nele Struck, Thomas Gärtner, Tilo Kircher, Eva-Lotta Brakemeier

**Affiliations:** ^1^Department of Clinical Psychology and Psychotherapy, University of Marburg, Marburg, Germany; ^2^Schoen Clinic Bad Arolsen, Bad Arolsen, Germany; ^3^Department of Psychiatry and Psychotherapy, University of Marburg, Marburg, Germany; ^4^Department of Clinical Psychology and Psychotherapy, University of Greifswald, Greifswald, Germany

**Keywords:** social cognition, childhood maltreatment, persistent depressive disorder, interpersonal problems, empathy

## Abstract

**Objective:** Little is known about the specific psychological features that differentiate persistent depressive disorder (PDD) and episodic depression (ED). Thus, the present study aimed to investigate differences in social cognition and interpersonal problems between these two forms of depression and healthy controls. In addition, we aimed to examine childhood maltreatment (CM) as a possible origin of these alterations.

**Methods:** In a cross-sectional study, adult patients with a current PDD (*n* = 34) or in a current episode of ED (*n* = 38), and healthy controls (*n* = 39) completed questionnaires about depression severity, empathy, interpersonal problems, and CM, as well as tests of affective theory of mind and facial emotion recognition.

**Results:** Patients with PDD reported higher empathic distress than patients with ED and healthy controls. Both depressive groups recognized angry faces with higher accuracy and reported more interpersonal problems, with no differences between PDD and ED. Empathic distress and interpersonal problems mediated the link between CM and depression in the combined sample.

**Limitations:** Patient groups were not drug-naïve and antidepressant intake might have influenced social-cognitive functions. Self-report measures of empathy and interpersonal problems are vulnerable to bias. The cross-sectional design does not allow causal conclusions.

**Conclusion:** Depressed patients may not show deficits in decoding the affective states of others and in feeling with others. However, depressed individuals—in particular patients with PDD—may feel easily overwhelmed by emotionally tense situations, resulting in empathic distress and avoidant/submissive interpersonal behavior. Exposure to CM might be an origin of alterations in social cognition and interpersonal problems.

## Introduction

According to the DSM-5 diagnostic criteria, a persistent depressive disorder (PDD) is characterized by symptoms of depressed mood for at least 2 years (1). Approximately 30% of depressed individuals develop a chronic course of the disorder, as defined by the PDD criteria (2). PDD is associated with an earlier age of onset, higher rates of comorbid mental and somatic disorders, more frequent suicide attempts, and higher treatment resistance when compared with episodic depression (ED) (3). Since approximately 75–80% of chronically depressed patients were exposed to at least moderate to severe childhood maltreatment (CM) (4), exposure to abuse and neglect in childhood is assumed to be a major risk factor for the development of PDD. Previous research shows a dose-response relationship between CM and depression severity as well as an association between CM and chronicity of depression (5). However, studies comparing the prevalence of CM in PDD and ED are rare and resulted in inconsistent findings (3, 6, 7).

In his interpersonal model of chronic depression, James McCullough — founder of the Cognitive Analysis System of Psychotherapy (CBASP) — describes pervasive interpersonal fear-avoidance and a perceptual disconnection from the interpersonal environment as the core psychopathology of PDD patients (8). He argues that specific theory of mind and empathy deficits in chronically depressed patients are rooted in early adverse relational experiences (9). His model also proposes that the interpersonal fear-avoidance in patients with PDD is characterized by a hostile-submissive interpersonal style, developed as an adaptation to a hostile, abusive, and neglectful environment in childhood. This behavior, in turn, deprives them of positive interpersonal experiences which contributes to the development and maintenance of depressive symptoms. There is good evidence for the efficacy of CBASP in the treatment of PDD (10, 11) and it is widely used to treat chronic depression, however, there is a lack of studies that comprehensively examine the underlying theoretical model.

### Social Cognition in Episodic and Persistent Depression

The term *theory of mind* (ToM) is defined as the cognitive ability to attribute mental states to oneself and others (12). While cognitive ToM refers to the attribution of thoughts and intention, affective ToM refers to the attribution of emotions (13). The ToM concept is overlapping with the term *perspective-taking* which has been described as the capacity to understand others' viewpoints and to consider these viewpoints when solving interpersonal problems (14). *Empathy* is defined as a multidimensional construct (14): the cognitive dimension of empathy is mostly overlapping and interchangeably used with the affective ToM concept while the affective dimension can be defined as the degree to which someone responds emotionally to the feelings of another person (15). Affective empathy may elicit (a) *empathic distress* which refers to aversive and self-oriented responses of personal anxiety and stress (14, 16) or (b) *empathic concern* which refers to other-oriented feelings of concern and warmth, facilitating pro-social behavior (14).

The most consistent finding in a review of empathy in adults with depressive symptoms was a link between depression and high levels of empathic distress (15). Results of another recent meta-analysis indicated that patients with depression show deficits in ToM and that the magnitude of these deficits is linked to depression severity (17). However, to our knowledge, only three studies to date have compared patients with PDD and ED in measures of empathy or ToM. Van Randenborgh et al. (7) and Ladegaard et al. (18) found no differences between patients with PDD and ED in self-report and objective measures of ToM. In the third study, patients with PDD reported more empathic distress than patients with ED and healthy controls (19). Depressed patients reported more difficulties in perspective-taking, with no differences between PDD and ED. No differences were found regarding empathic concern (19). Further studies are needed to clarify whether there are differences between ED and PDD in terms of empathy and ToM and, if so, in which specific domains they occur.

The ability to recognize emotions correctly is essential for positive interactions with others. Dalili et al. (20) report in their meta-analysis impaired emotion recognition in patients with depression for all emotions except for sadness. Other studies indicate that depressed patients have a negative response bias or lack a positive response bias compared with healthy controls, in particular when ambiguous or neutral faces are presented [e.g., (21–24)]. This bias to misinterpret faces as negative could contribute to the development and maintenance of depressive symptoms. To our knowledge, no study so far has investigated differences between ED and PDD with regard to emotion recognition biases.

### Interpersonal Problems in Episodic and Persistent Depression

According to the Interpersonal Circumplex Model (25), all interpersonal behavior can be classified in two-dimensional space on the axes *affiliation* and *dominance*. A recent meta-analysis supports McCullough's (8) assumption of elevated submissiveness, hostility, and hostile-submissiveness in patients with PDD and, to a smaller degree, in patients with ED (26). However, to date, only very few studies directly compared the two patient groups. Constantino et al. found that patients with PDD and ED did not differ in submissiveness, friendly-submissiveness, or hostile-submissiveness, but they differed in levels of hostility (27). A recent study also indicates higher levels of specific interpersonal skill deficits (peroperational thinking) in patients with PDD when compared with ED and an association between these deficits and depression severity over the course of 2 years (28).

### Childhood Maltreatment, Social Cognition, and Interpersonal Problems

CM has been consistently identified as a major risk factor for the development of a lifetime diagnosis of a major depression (5) and, as described above, possible mediators of this relationship are alterations in social cognition and interpersonal behavior (9, 29).

A negative impact of CM on affective ToM performance has been shown in several samples, e.g., in a large online convenience sample (30), and in patients with borderline personality disorder (31). Two recent studies investigated the link between CM and affective ToM in adult patients with depression (32, 33). Both studies found a link between emotional abuse and deficits in affective ToM. Regarding emotion recognition, previous studies suggest a general impairment in maltreated children (34). However, there is also evidence for a threat bias in abused children and young adults who recognized anger at a lower emotion intensity when compared with controls (35–37). There is a lack of studies investigating the relationship between CM and emotion recognition accuracy and biases in patients with depression (38).

Previous research also suggests an association between CM and interpersonal problems (39–41) and a recent study indicates that interpersonal fears mediate the effect of CM on specific interpersonal skill deficits (42). However, most studies to date have used healthy college samples, so that more findings on the relationship between CM and interpersonal problems in patients with depression are needed.

### Aims of the Study

In the current study, we aim to test some of McCullough's theoretical views empirically. First, we aim to examine differences in social cognition between patients with PDD and ED and healthy controls. Based on the literature mentioned above, we expect impaired affective ToM abilities and higher levels of empathic distress (a) in patients with PDD when compared with patients with ED and (b) in both depressed groups when compared with healthy controls. We also hypothesize a negative emotion recognition bias in patients with depression. We expect that both patient groups recognize more sadness and anger and less happiness in morphed faces. Second, we aim to compare interpersonal problems between groups. Based on the previous research findings, we hypothesize (a) higher levels of submissiveness in all patients with depression when compared with healthy controls and (b) higher levels of hostile-submissiveness in patients with PDD when compared with patients with ED and healthy controls. Finally, we aim to investigate CM as a possible origin of these alterations. We expect higher levels of CM in individuals with PDD when compared with patients with ED and healthy controls. We hypothesize a link between CM and deficits in ToM, increased empathic distress, increased negative emotion recognition bias, and increased interpersonal hostility and submissiveness in the combined sample. Finally, we will explore if social cognitive variables and interpersonal problems mediate the link between CM and depression severity in the combined sample.

## Materials and Methods

### Participants

The sample of the present cross-sectional study consisted of 111 individuals: 38 patients with an ED, 34 patients with a PDD, and 39 healthy control participants. The ethics committees of the Department of Medicine and the Department of Psychology at the University of Marburg approved the protocol. Patients were recruited from one outpatient and two inpatient facilities through invitations to participate (e.g., after psychoeducational lectures or via flyers). Healthy controls were recruited via advertisements in regional newspapers, notices in public places, and online advertisements. Participants received financial compensation. Written informed consent was obtained from all participants. General inclusion criteria were an age between 18 and 65 and adequate German language skills. The healthy control group additionally met the following criteria: no current mental disorder assessed by the Structured Clinical Interview for DSM-IV Interview (SCID) (43) and no diagnosed mental disorder in the last 10 years according to self-report. Patients were included if they met either criteria for a current major depressive disorder (duration < 24 months, ED group) or criteria for a current persistent depressive disorder (duration ≥ 24 months, PDD group) according to DSM-5 criteria (1). This was assessed by SCID interviews and an additional interview using a life chart covering the last 24 months [based on (44)]. Participants were excluded if they met any of the following criteria: acute suicidality, a diagnosis of schizophrenia or bipolar disorder, dementia, or severe cognitive impairments. A total of 119 participants were assessed for eligibility of which eight were excluded: five patients because they no longer met criteria for a current episode of ED or PDD and three patients because of missing data/incomplete study participation, resulting in the final sample of *N* = 111. Due to difficulties in data collection, emotion recognition data was missing from seven of the subjects. After screening for outliers of the emotion recognition data, two healthy subjects were excluded for the emotion recognition analyses because of strong evidence of careless responding. Further individual outliers were considered valid answers and therefore not excluded. This resulted in a reduced sample of 102 individuals for the emotion recognition analyses (35 ED, 30 PDD, 37 HC).

The demographic and clinical characteristics of the three groups are presented in Table 1. Briefly, groups did not differ with respect to age, gender, and years of education. When comparing patients with ED and PDD, there were no significant differences with respect to the age of onset, number of inpatient and outpatient treatments, and the use of antidepressants. The three groups differed with regard to depression severity, with the highest scores in the PDD group, followed by the ED group, and the lowest scores in the healthy control group. Repeating the comparison of demographic and clinical characteristics between groups in the reduced sample for the emotion recognition analyses yielded in the same results, with the exception that the ED and PDD group differed in the use of antidepressants, with significantly higher use in the PDD group (ED = 51.4%, PDD = 76.7%).

**Table 1:** Demographic and clinical characteristics of the sample.

	**HC (*****n*** **=** **39)**		**ED (*****n*** **=** **38)**		**PDD (*****n*** **=** **34)**		**Test statistic *F*/*t*/**χ**^2^**	***p***
**Characteristic**	***M***	***SD***	***M***	***SD***	***M***	***SD***		
Age	39.92	14.93	41.63	12.76	44.85	12.98	^a^1.21	0.3
% Female	53.80%		50.00%		55.90%		^b^0.26	0.88
Years of education	14.26	2.23	13.53	2.09	13.5	2.36	^a^1.41	0.25
% Married/	30.80%		52.60%		44.10%		^b^3.83	0.15
in partnership								
Age of onset	–	–	30.45	14.55	25.21	14.35	^c^1.52	0.13
Number outpatient treatments	–	–	1.87	3.84	4.26	9.46	^c^−1.44	0.16
Number inpatient treatments	–	–	1.61	1.64	1.65	1.48	^c^−0.11	0.91
% Antidepressants	–		55.30%		76.50%		^b^3.56	0.06
Depression (BDI–II)	3.95	4.23	25.95	12.68	33.79	13.54	^d^116.25	**<0.001**

*CTQ, Childhood Trauma Questionnaire; HC, healthy control group; ED, episodic depression; PDD, persistent depressive disorder; BDI-II, Beck Depression Inventory*.

a *ANOVA*.

b *Chi-Square Test*.

c *t-Test*.

d *Welch-ANOVA*.

*Bold values are statistically significant with p < 0.05*.

The 34 patients with PDD had the following subtypes of PDD: *n* = 1 (2.9%) with pure dysthymic syndrome; *n* = 15 (44.1%) with persistent major depressive episode; *n* = 16 (47.1%) with intermittent major depressive episode, with current episode; *n* = 2 (5.9%) with intermittent depressive episode, without current episode.

### Measures

#### Beck Depression Inventory-II (BDI-II)

The severity of depressive symptoms was measured by self-report using the Beck Depression Inventory, assessing depressive symptoms in the last 2 weeks with 21 Items [BDI-II, (45); German version: (46)]. The internal consistency of the BDI-II was between α = 0.84 and α = 0.90 in a previous study (47).

#### Childhood Trauma Questionnaire (CTQ)

CM was assessed by retrospective self-report with the 28-item version of the Childhood Trauma Questionnaire [CTQ-SF; (48), German version: (49)]. The CTQ measures five types of CM: emotional abuse (α = 0.87), physical abuse (α = 0.83), sexual abuse (α = 0.96), emotional neglect (α = 0.89), and physical neglect (α = 0.61, all α in this sample). The response options range from 1 (= *never true*) to 5 (= *very often true*).

#### Interpersonal Reactivity Index (IRI)

A shortened and validated German version of the interpersonal reactivity index (IRI) self-report survey was used to measure dispositional empathic traits in four subscales [(50); German version: (51)]. The *perspective-taking* subscale assesses spontaneous attempts to adopt the perspectives of other people and see things from their point of view (α = 0.78); the *empathic concern* subscale assesses feelings of warmth, compassion, and concern for others when confronted with negative experiences of others (α = 0.76); the *personal distress* subscale (synonym for *empathic distress*) measures personal feelings of anxiety and discomfort resulting from observing another's negative experiences (α = 0.78); and the *fantasy* subscale assesses the tendency to identify with characters in movies, novels, plays and other fictional situations (α = 0.73, all α in this sample) (50). The shortened German version consists of four items per scale (51).

#### Reading the Mind in the Eyes Test (RMET)

The revised version of the Reading the Mind in the Eyes Test (RMET) was used to measure affective ToM (52). In this test, subjects are presented with 36 black-and-white photographs only showing the eye region of faces. Four attributes (e.g., serious, ashamed, alarmed, and bewildered) are displayed around the eyes and subjects are asked to choose the word that matches the person's mental state best. The total number of errors was counted, as well as separate error sums for pictures with positive valence (9 items), negative valence (12 items), and neutral valence (15 items) based on a valence analysis by Kometer et al. (53).

#### Facial Expression Recognition Task (FERT)

Emotion recognition was assessed with the facial expression recognition task previously described (54). For this task, pictures of facial expressions presenting the six basic emotions happiness, sadness, fear, anger, surprise, and disgust were taken from the Ekman and Friesen Pictures of Affect Series (55) and were morphed between each prototype (100%) and neutral (0%) in 10% steps. A total of 250 stimuli were presented: four examples of each emotion at each intensity and 10 neutral faces. Each stimulus was presented for 500 ms and then replaced by a blank screen. Subjects were asked to give their response as quickly and accurately as possible by pressing one of the seven labeled keys on a response box.

#### Inventory of Interpersonal Problems (IIP)

The German short version of the Inventory of Interpersonal Problems (IIP) was used to assess self-reported interpersonal problems in 32-items (56). The scale is based on the Interpersonal Circumplex Model which describes all interpersonal behavior in a two-dimensional space along the two main axes *affiliation* and *dominance* (25). The IIP measures eight domains of interpersonal problems: behavior that is overly, 1. domineering/controlling (PA), 2. vindictive/self-centered (BC), 3. cold/distant (DE), 4. socially inhibited/avoidant (FG), 5. nonassertive (HI), 6. accommodating/exploitable (JK), 7. self-sacrificing/nurturant (LM), 8. intrusive/needy (NO). The dimension cold/distant (DE) corresponds to hostile interpersonal behavior, socially inhibited/avoidant (FG) to hostile-submissive, and nonassertive (HI) to submissive behavior in McCullough's model (9). The German version of the IIP-32 has shown good psychometric properties (57). In the current sample, Cronbach's alpha of the total IIP score was 0.90, alphas of the relevant scales ranged from 0.69 (JK) to 0.82 (FG).

### Statistical Analyses

Statistical Analyses were conducted using IBM SPSS Statistics 22.0. Scale means were calculated if at least 75% of the items were answered. Group differences regarding demographic and clinical characteristics, social cognitive variables, interpersonal problems, and CM were assessed using one-way independent analyses of variance (ANOVA). Welch-Tests were applied in case of unequal variances. *Post*-*hoc* tests were Bonferroni-corrected for multiple comparisons. To test the hypothesized socio-developmental origin of differences in social cognition and interpersonal behaviors, associations between CM and ToM, empathy, interpersonal problems, and depression were explored with partial correlations controlled for age and gender. Next, to examine the hypothesized mediation with CM as the independent variable, social-cognitive variables as mediators and depression severity as dependent variable, a mediation analysis using the PROCESS Macro [(58); Model 4] for SPSS was performed. Only socio-cognitive variables related to CM and depression in the correlational analyses were included as mediators (explorative selection of relevant mediators). To test the statistical significance of the indirect effects, we used bias-corrected 95% bootstrap confidence intervals based on 5,000 bootstrap samples.

## Results

### Between-Group Differences in Social Cognition

The statistics and effect sizes of the comparison of empathy, ToM, emotion recognition accuracy, and interpersonal problems between groups are presented in Table 2.

**Table 2:** Comparison of social cognition and interpersonal problems between groups.

	**Group**							**Effect size**		
	**HC (*****n*** **=** **39)**		**ED (*****n*** **=** **38)**		**PDD (*****n*** **=** **34)**		**Test statistic**	**HC vs. ED**	**HC vs. PDD**	**ED vs. PDD**
**Characteristic**	***M***	***SD***	***M***	***SD***	***M***	***SD***	***F_***2,108***_***	***d***	***d***	***d***
**Empathy (IRI)**										
Empathic concern	3.39	0.79	3.84	0.64	3.93	0.56	6.851^**^	0.63^*^	0.79^**^	0.15
Perspective taking	3.79	0.73	3.32	0.74	3.51	0.74	3.881^*^	−0.64^*^	−0.38	0.26
Empathic distress	2.3	0.61	3.15	0.66	3.56	0.85	30.347^***^	1.34^***^	1.70^***^	0.54^*^
**Affective ToM (RMET)**										
Total error	12.69	4.61	12.68	5.06	11.74	3.54	0.534	<0.01	−0.23	−0.22
**Emotion Recognition Accuracy (FERT)**^***a***^										
Anger^a^	50.95	19.16	61.64	11.61	60.17	9.26	^c^4.31^*^	0.67^**^	0.61^*^	−0.14
Sadness^a^	56.35	17.03	58.64	16.6	61.92	12.61	1.04	0.14	0.37	0.22
Happiness^a^	78.78	7.85	76.64	8.11	77.5	8.2	0.65	−0.27	−0.16	0.11
Global^a^	56.58	10.53	58.7	8.29	59	5.29	^c^0.75	0.22	0.29	0.04
**Interpersonal Problems (IIP)**										
IIP–total^b^	1.2	0.5	1.9	0.44	2.07	0.41	37.231^***^	1.49^***^	1.90^***^	0.4
Hostile/DE	0.76	0.75	1.61	0.85	1.75	0.83	16.491^***^	1.06^***^	1.25^***^	0.17
Hostile–submis./FG	1.12	0.8	2.12	0.72	2.45	1.04	^c^24.318^***^	1.31^***^	1.43^***^	0.37
Submissive/HI	1.71	0.86	2.24	0.8	2.72	0.97	12.099^***^	0.64^*^	1.10^***^	0.54
Friendly–submis./JK	1.75	0.82	2.49	0.79	2.71	0.77	14.937^***^	0.92^***^	1.21^***^	0.28

*CTQ, Childhood Trauma Questionnaire; HC, healthy control group; ED, episodic depression; PDD, persistent depressive disorder; IRI, Interpersonal Reactivity Index; RMET, Reading the Mind in the Eyes Test; FERT, Facial Expression Recognition Task; IIP, Inventory of Interpersonal Problems; DE, cold/distant; FG, socially inhibited; HI, nonassertive; JK, accommodating*.

a *N = 102 (HC n = 37, ED n = 35, PDD n = 30)*.

b *n = 107*.

c *Welch–ANOVA; Bonferroni Pos–hoc Tests for all comparisons*.

* *p < .05*,

** *p < .01*,

*** *p < .001*.

Regarding empathic distress, groups differed significantly. Bonferroni-corrected *post-hoc* tests revealed that patients with PDD reported significantly more empathic distress compared to healthy controls and patients with ED. The difference between healthy controls and patients with ED was also statistically significant. Regarding empathic concern, groups also differed significantly. Patients with PDD and ED reported significantly more empathic concern compared with healthy controls, with no significant difference between patients with PDD and ED. Regarding perspective-taking, groups also differed significantly. Patients with ED reported significantly less perspective-taking when compared with healthy controls. There were no differences in reported perspective-taking between patients with PDD when compared with healthy controls or patients with ED. The three groups did not differ with respect to RMET errors (see Table 2). Even when the valences (positive, negative, neutral) were considered separately, there were no significant differences between patients with ED, PDD, and healthy controls in any valence of the RMET (see Supplementary Material 1).

Patients with ED and PDD recognized angry emotional expressions with higher accuracy than healthy controls. The diagnostic groups did not differ in the recognition of happiness, sadness, and global emotion recognition. These results did not change when we included the use of antidepressants as a covariate. Further analyses of differences in accuracy and reaction times for recognition of all facial expressions are presented in Supplementary Material 2.

### Between-Group Differences in Interpersonal Problems

Regarding interpersonal problems, there were significant differences between groups (see Table 2). With respect to the IIP total score and all examined subscales, patients with ED and PDD reported significantly more interpersonal problems when compared with healthy controls. Patients with ED and PDD did not differ significantly in any of the examined subscales or the total IIP. See Supplementary Material 1 for the IIP subscales not considered in our hypothesis.

### Between-Group Differences in Childhood Maltreatment

The statistics and effect sizes of the prevalence of different types of CM in the three groups are presented in Table 3. The groups differed in the CTQ total score and all subscales of the CTQ. Patients with PDD reported more CM of all types when compared with healthy controls. They also reported more emotional abuse, physical abuse, and higher total CM than patients with ED. Patients with ED reported increased levels of emotional abuse, emotional neglect, and total CM when compared with healthy controls.

**Table 3:** Comparison of self-reported childhood maltreatment between groups.

	**Group**							**Effect size**		
	**HC (*****n*** **=** **39)**		**ED (*****n*** **=** **38)**		**PDD (*****n*** **=** **34)**		**Test statistic**	**HC vs. ED**	**HC vs. PDD**	**ED vs. PDD**
**Characteristic**	***M***	***SD***	***M***	***SD***	***M***	***SD***	***F_***2,108***_***	***d***	***d***	***d***
CTQ total score	34.97	9.77	43.63	11.95	53.26	19.76	^a^14.43^***^	0.79^*^	1.17^***^	0.59^*^
										
Emotional abuse	7.56	2.82	10.76	4.86	13.71	5.45	^a^19.96^***^	0.81**	1.42^***^	0.57^*^
Physical abuse	5.9	2.1	6.47	2.24	9.21	4.92	^a^6.61^**^	0.26	0.88^***^	0.72^**^
Sexual abuse	5.26	0.79	5.63	1.58	7.35	5.34	^a^3.23^*^	0.3	0.55^*^	0.44
Emotional neglect	9.77	4.63	12.82	4.75	14.03	5.21	^b^7.60^**^	0.65^*^	0.86^**^	0.24
Physical neglect	6.49	1.89	7.95	3.24	8.97	3.76	^a^7.45^**^	0.55	0.83^**^	0.29

*CTQ, Childhood Trauma Questionnaire; M, mean; SD, standard deviation; HC, healthy control group; ED, episodic depression; PDD, persistent depressive disorder*.

a *Welch-ANOVA*.

b *ANOVA; Bonferroni Post-hoc Tests*.

* *p < 0.05*,

** *p < 0.01*,

*** *p < 0.001*.

### Associations Between CM, Social Cognition, and Interpersonal Problems and Test of Mediation

Partial correlations between CM, empathy variables, emotion recognition accuracy, interpersonal problems, and depression severity, controlled for age and gender in the full sample are presented in Table 4. CM was positively correlated with depression severity with large effect size and with empathic distress and interpersonal problems with medium to large effect size. There was a small to medium negative correlation between CM and the recognition of happiness, which can be interpreted as a trend (*p* = 0.055). Depression severity correlated with large effect size positively with empathic distress and interpersonal problems, with medium to large effect size positively with empathic concern, and with small to medium effect size positively with the recognition accuracy of anger. Bivariate correlations are presented in Supplementary Material 3 and partial correlation between CM and different facets of interpersonal problems in Supplementary Material 4. CM correlated with all subscales of the IIP, apart from too domineering/controlling and too vindictive/self-centered interpersonal behavior. CM was most strongly associated with socially inhibited/avoidant behavior (*r* = 0.41, *p* < 0.001).

**Table 4:** Partial correlations between childhood maltreatment, social cognitive variables, interpersonal problems, and depression, controlled for age and gender.

**Variable**	**1**	**2**	**3**	**4**	**5**	**6**	**7**	**8**	**9**	**10**
1. Childhood Maltreatment	1									
2. Empathic Concern	0.18	1								
3. Perspective Taking	−0.17	0.28^**^	1							
4. Empathic Distress	0.45^***^	0.24^*^	−0.20^*^	1						
5. RMET errors	−0.08	−0.13	−0.06	−0.02	1					
6. Anger accuracy^a^	0.11	0.12	−0.11	0.17	−0.16	1				
7. Happiness accuracy^a^	−0.20^†^	−0.07	0.03	−0.26^**^	−0.21^*^	0.13	1			
8. Sadness accuracy^a^	0.14	0.03	0.06	0.07	−0.25^*^	0.29^**^	0.19	1		
9. FERT global accuracy^a^	0.02	0.18	0.11	0.05	−0.34^**^	0.65^***^	0.37^***^	0.60^***^		
10. Interpersonal Problems^b^	0.43^***^	0.21^*^	−0.33^**^	0.75^***^	0.03	0.11	−0.14	0.12	−0.01	1
11. Depression	0.52^***^	0.35^***^	−0.18	0.73^***^	−0.02	0.22^*^	−0.12	0.19	0.11	0.75^***^

*RMET, Reading the Mind in the Eyes Test; FERT, Facial Expression Recognition Task*.

† *p < 0.06*,

* *p < 0.05*,

** *p < 0.01*,

*** *p < 0.001*.

a *n = 102*;

b *n = 107*.

**Figure 1:**
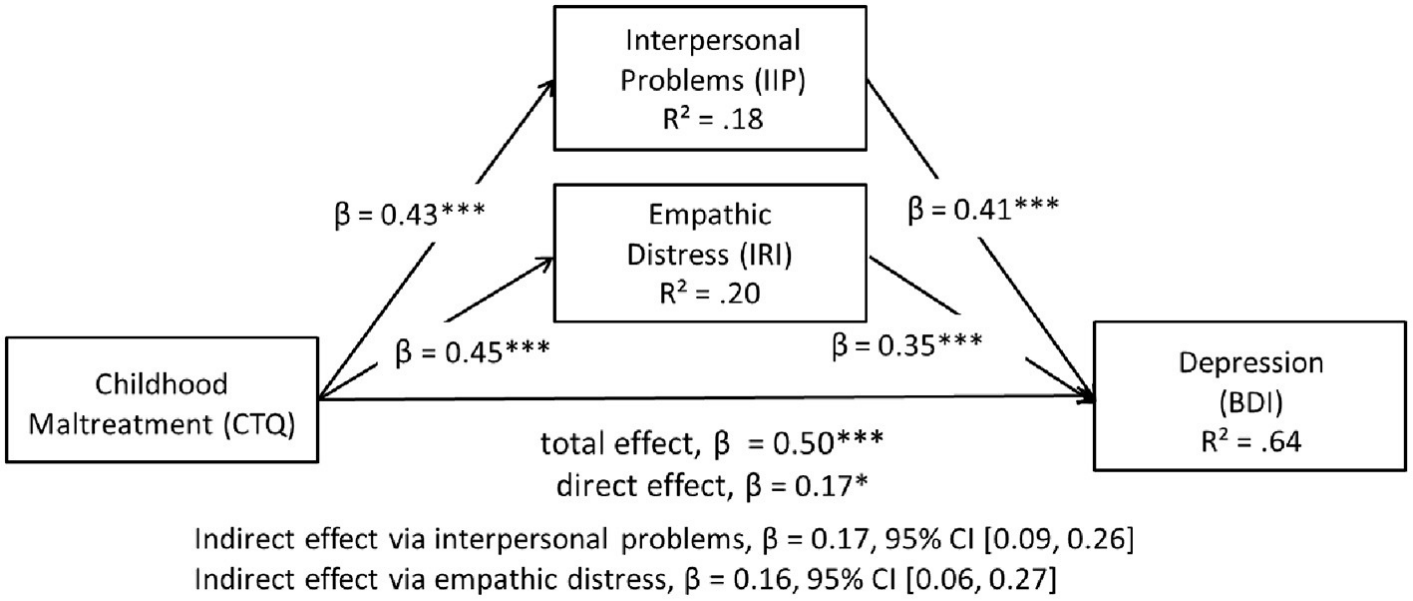
Model of childhood maltreatment as a predictor of depression severity mediated by interpersonal problems and empathic distress in the combined sample. Standardized coefficients are reported for each path. *p < 0.05, ***p < 0.001.

Based on these correlational findings, we examined a mediational model with empathic distress and interpersonal problems as mediators of the link between CM and depression severity in the combined sample. Results provided support for the hypothesized mediation model (Figure 1). There were significant indirect effects of CM on depression via interpersonal problems, β = 0.17, 95% CI [0.09, 0.26] and via personal distress, β = 0.16, 95% CI [0.06, 0.27]. The direct effect of CM on depression remained significant after including the mediators, β = 0.17, *p* = 0.01, supporting a partial mediation model.

## Discussion

### Social Cognition in Episodic and Persistent Depression

The first aim of the current study was to compare social cognition in patients with PDD, ED, and in healthy controls. As hypothesized, we found increased empathic distress in patients with PDD, followed by patients with ED and healthy controls. Interestingly, we also found increased empathic concern in both depressive groups. However, in contrast to our hypothesis, there were no differences in affective ToM between groups. In parts we could confirm the assumption of a negative emotion recognition bias in depression: both patient groups were more sensitive in the recognition of anger in faces; however, this was not the case for sadness and the two patient groups did not differ in the recognition of anger, sadness, or happiness.

Interestingly, our results indicate that depressed patients do not show deficits in decoding the affective states of others but that they have difficulties in handling another person's negative emotional state or suffering and might be overwhelmed by emotionally tense situations resulting in empathic distress. This feeling of empathic distress might be even more pronounced in patients with PDD compared with ED, which is in accordance with a previous study by Domes et al. (19). In fact, the higher empathic concern in the depressive groups and the correlation of empathic concern with depression severity also suggest that depressed patients might be even hypersensitive to the feelings of others which is in line with some previous findings and theories [(59, 60); however see also (15)]. Recent findings suggest that deficits in emotion regulation (61), high levels of alexithymia (62), and generalized guilt and shame (59) in depressed patients might result in high levels of affective empathy no longer having a protective effect. Under these conditions, high levels of affective empathy might even lead to a feeling of being overwhelmed and trigger empathic distress and depressive symptoms. More research on mechanisms and moderators regarding the relationship between affective empathy, emotional contagion, empathic distress, and depression is therefore needed.

Contrary to our hypothesis, we did not find any differences in affective ToM (as measured by the RMET) between groups, in neither of the depressive groups and for no valence. It is unlikely that this was due to low statistical power, as the effect sizes were small and contrary to our hypothesis (lowest error score in the PDD group) and we found no correlation between RMET errors and depression severity. Previous research comparing depressed patients with healthy controls in the RMET has been very inconsistent [e.g., (63–66)]. One possible explanation is that the depressive groups in the various studies differed in clinical or demographic variables. More moderator analyses are needed to explain the inconsistencies. It is also possible that the RMET is not sensitive enough to reliably detect a potential negative recognition bias. It should be noted, that the RMET is not a typical ToM test and has also been labeled as emotion recognition task instead (67). In contrast to the RMET results, we were able to show a negative recognition bias in the analyses of the emotion recognition data measured with the FERT which uses morphed images and thus has a variation in the emotional intensity of displayed facial expressions. In line with some previous findings [(21); however, see also (20)], patients with depression recognized anger with higher accuracy compared with healthy controls. Surprisingly, we found no bias in the recognition of sadness and no deficits in the recognition of facial expression with positive valence as in previous studies (21, 24). However, particularly with regard to the emotion recognition data, we need to discuss the statistical power to detect small effects (see below).

### Interpersonal Problems in Episodic and Persistent Depression

Our second aim was to compare interpersonal problems in patients with ED, PDD, and in healthy controls. We hypothesized (a) higher levels of submissiveness in all patients with depression when compared with healthy controls and (b) higher levels of hostile-submissiveness in patients with PDD when compared with patients with ED and healthy controls. Our results confirmed the first part of the hypothesis as both patient groups reported more interpersonal problems resulting from submissive behavior compared with the healthy control group. The effect size was medium for the ED group and large for the PDD group. This is in line with previous findings (26). However, the second part of the hypothesis could not be confirmed: patients with PDD did not report significantly more interpersonal problems resulting from hostile-submissive behavior than patients with ED. At a descriptive level, there was a trend for the PDD group to report more interpersonal problems, and the subscale on which the two depressive groups differed the most was the subscale of problems resulting from submissive behavior (non-significant, but medium effect size). This trend indicates that this difference between ED and PDD might be significant when replicated in a larger sample (see Limitations).

Interestingly, interpersonal problems corresponding to hostile and submissive behavior were strongly correlated with empathic distress, while there was no association with affective ToM and emotion recognition abilities. Based on these findings we argue that the experience of empathic distress could strengthen fears of interaction with others and might lead to a more avoidant interpersonal style, while deficits and biases in decoding emotions might play a less prominent role in the development of interpersonal problems than previously assumed. The causal relationship between empathic distress and interpersonal problems could also be bidirectional, in the form that a lack of interpersonal skills leads to a faster overload in difficult situations resulting in empathic distress.

### Childhood Maltreatment as an Origin of Alteration in Social Cognition and Interpersonal Problems

Our third aim was to examine CM as a possible origin of these alterations and to test a mediation model with CM as independent variable, social cognition and interpersonal problems as mediators, and depressive symptoms as outcome. Patients with PDD reported more CM of all types when compared with healthy controls, and more physical abuse, emotional abuse, and higher general CM levels when compared with patients with ED. As hypothesized, CM was associated with increased depression severity, empathic distress, and interpersonal problems. However, there was no association with affective ToM abilities. At a trend level, CM was negatively associated with the recognition of happiness in faces. Results of the hypothesized mediation model suggest that interpersonal problems and empathic distress mediate the link between CM and depression.

Our findings suggest that the alterations in empathy and interpersonal problems in depressed patients might be partially rooted in a history of exposure to CM. It has been argued that CM can lead to changes in social cognition in two ways: (a) via a lack of learning and developmental opportunities due to a lack of positive stimulation (neglect) and (b) via a sensitization to threat-relevant stimuli as an adaptation to the repeated exposure to threat (abuse) (68).

Consistent with earlier findings in non-clinical samples (39, 41), CM was linked with interpersonal problems and empathic distress, and the association between CM and depression severity was mediated by interpersonal problems and empathic distress. This finding also supports McCullough's theoretical model (9), proposing that depressed patients who were exposed to histories of CM show pervasive interpersonal fear-avoidance resulting in dysfunctional interpersonal behavior. Possibly, those interpersonal problems lead to higher depression severity via lower perceived social support and weaker social ties (69, 70). However, contrary to our hypothesis, CM and depression severity were not associated with general deficits in affective ToM. Taken together, CM was not associated with difficulties in decoding affective states of others, but with a feeling of being overwhelmed by negative affective states of others.

### Limitations

Some limitations of the current study should be noted. First, we used self-report measures of empathic abilities and interpersonal problems which might be state-dependent and biased by social desirability. It has also been argued, that socio-cognitive deficits in depressed patients might not be detectable with laboratory tests because they are not comparable with daily interpersonal interactions in which the participant is directly and actively involved (71). Therefore, further studies should develop and use more objective and behavioral measures with participants ideally being actively involved themselves. Another limitation is that our depressed sample was diverse regarding the intake of antidepressants with differences between the ED and PDD groups. Previous studies showed that antidepressant administration might ameliorate the negative emotion recognition bias (54) and reduce emotional contagion when confronted with the pain of others (72). Thus, the effects of antidepressants could have led to an underestimation of the differences between groups regarding biases in emotion recognition and empathic distress. However, controlling for the use of antidepressants in our emotion recognition analyses did not change the results. More studies investigating social cognition in drug naïve samples are needed. A further limitation is the cross-sectional design of the study which does not allow to draw causal conclusions. Although the hypothesized temporal sequence of exposure to CM, social cognitive functioning/interpersonal problems, and clinical outcome in the mediation model is theoretically plausible, a reverse order cannot be excluded: e.g., symptoms of depression could influence interpersonal submissiveness or empathic distress. Therefore, the mediation analysis should be interpreted with caution and more longitudinal studies are needed. As the RMET only measures a small facet of ToM, overlapping with the concept of emotion recognition, further studies should include more tests covering other aspects of ToM, e.g., also the cognitive dimension. Limitations regarding the statistical power to detect small effect sizes—especially regarding expected small biases in facial emotion recognition and regarding the differences between ED and PDD in interpersonal problems—should also be mentioned. We must, therefore, be careful with statements regarding effects that we have not been able to show in this study.

### Practical Implications

Applying these results to the treatment of depression in general and of PDD in particular, emphasizes the importance of practical interpersonal skill training, as implemented e.g. in CBASP situational analyses using role plays. As depressed patients appear to have no deficits in “feeling with” others (rather may even do so more strongly) but to deal with their own feelings resulting from this, our findings also suggest a therapeutic focus on emotion regulation abilities. A focus on emotion regulation abilities corresponds to psychotherapeutic strategies in the Dialectic Behavior Therapy [DBT; (73)] for the treatment of Borderline Personality Disorders, another disorder characterized by a very high prevalence of histories of CM exposure (6). Once more, the results of this study highlight the outstanding importance of efforts to prevent CM and programs to support maltreated children and adolescence to reduce further consequences as the risk of chronic mental illness.

## Data Availability Statement

The raw data supporting the conclusions of this article will be made available by the authors, without undue reservation.

## Ethics Statement

The studies involving human participants were reviewed and approved by the Department of Psychology University of Marburg and the Department of Medicine University of Marburg. The patients/participants provided their written informed consent to participate in this study.

## Author Contributions

NS and E-LB planned the study. NS conducted the statistical analyses and drafted the manuscript. NS, TG, TK, and E-LB all contributed to organizing data collection, providing feedback, and revising the manuscript. All authors contributed to the article and approved the submitted version.

## Conflict of Interest

The authors declare that the research was conducted in the absence of any commercial or financial relationships that could be construed as a potential conflict of interest.

## References

[1] American Psychiatric Association Diagnostic and Statistical Manual of Mental Disorders (DSM-5®). Washington, DC: American Psychiatric Pub (2013).

[2] Murphy JA Byrne GJ. Prevalence and correlates of the proposed DSM-5 diagnosis of chronic depressive disorder. J Affect Disord. (2012) 139:172–80. 10.1016/j.jad.2012.01.033 22381955

[3] Köhler S Chrysanthou S Guhn A Sterzer P. Differences between chronic and nonchronic depression: systematic review and implications for treatment. Depress Anxiety. (2019) 36:18–30. 10.1002/da.22835 30300454

[4] Brakemeier E-L Radtke M Engel V Zimmermann J Tuschen-Caffier B Hautzinger M. Overcoming treatment resistance in chronic depression: a pilot study on outcome and feasibility of the cognitive behavioral analysis system of psychotherapy as an inpatient treatment program. Psychother Psychosom. (2015) 84:51–6. 10.1159/000369586 25547778

[5] Nelson J Klumparendt A Doebler P Ehring T. Childhood maltreatment and characteristics of adult depression: meta-analysis. Br J Psychiatry. (2017) 210:96–104. 10.1192/bjp.bp.115.180752 27908895

[6] Brakemeier EL Dobias J Hertel J Bohus M Limberger MF Schramm E. Childhood maltreatment in women with borderline personality disorder, chronic depression, and episodic depression, and in healthy controls. Psychother Psychosom. (2018) 87:49–51. 10.1159/000484481 29306947

[7] Van Randenborgh A Hüffmeier J Victor D Klocke K Borlinghaus J Pawelzik M. Contrasting chronic with episodic depression: an analysis of distorted socio-emotional information processing in chronic depression. J Affect Disord. (2012) 141:177–84. 10.1016/j.jad.2012.02.039 22520739

[8] McCullough JP Jr Schramm E Penberthy JK CBASP as a Distinctive Treatment for Persistent Depressive Disorder: Distinctive Features. New York, NY: Routledge (2015).

[9] McCullough JP Jr. Treatment for chronic depression using cognitive behavioral analysis system of psychotherapy (CBASP). J Clin Psychol. (2003) 59:833–46. 10.1002/jclp.10176 12858425

[10] Jobst A Brakemeier EL Buchheim A Caspar F Cuijpers P Ebmeier KP. European Psychiatric Association Guidance on psychotherapy in chronic depression across Europe. Eur Psychiatry. (2016) 33:18–36. 10.1016/j.eurpsy.2015.12.003 26854984

[11] Negt P Brakemeier E-L Michalak J Winter L Bleich S Kahl KG. The treatment of chronic depression with cognitive behavioral analysis system of psychotherapy: a systematic review and meta-analysis of randomized-controlled clinical trials. Brain Behav. (2016) 486:e00486. 10.1002/brb3.486 27247856 PMC4864084

[12] Premack D Woodruff G Does the chimpanzee have a theory of mind? Behav Brain Sci. (1978) 1:515–26. 10.1017/S0140525X00076512

[13] Shamay-Tsoory SG Shur S Barcai-Goodman L Medlovich S Harari H Levkovitz Y. Dissociation of cognitive from affective components of theory of mind in schizophrenia. Psychiatry Res. (2007) 149:11–23. 10.1016/j.psychres.2005.10.018 17107716

[14] Davis MH A mulitdimensional approach to individual differences in empathy. Pers Soc Psychol. (1983) 44:113–26. 10.1037/0022-3514.44.1.113

[15] Schreiter S Pijnenborg GHM aan het Rot M Empathy in adults with clinical or subclinical depressive symptoms. J Affect Disord. (2013) 150:1–16. 10.1016/j.jad.2013.03.009 23668900

[16] Singer T Klimecki OM Empathy and compassion. Curr Biol. (2014) 24:R875–78. 10.1016/j.cub.2014.06.054 25247366

[17] Bora E Berk M. Theory of mind in major depressive disorder: a meta-analysis. J Affect Disord. (2016) 191:49–55. 10.1016/j.jad.2015.11.023 26655114

[18] Ladegaard N Lysaker PH Larsen ER Videbech P. A comparison of capacities for social cognition and metacognition in first episode and prolonged depression. Psychiatry Res. (2014) 220:883–9. 10.1016/j.psychres.2014.10.005 25453639

[19] Domes G Spenthof I Radtke M Isaksson A Normann C Heinrichs M. Autistic traits and empathy in chronic vs. episodic depression. J Affect Disord. (2016) 195:144–7. 10.1016/j.jad.2016.02.006 26895092

[20] Dalili MN Penton-Voak IS Harmer CJ Munafo MR. Meta-analysis of emotion recognition deficits in major depressive disorder. Psychol Med. (2015) 45:1135–44. 10.1017/S0033291714002591 25395075 PMC4712476

[21] Bomfim AJL Ribeiro RA Chagas MHN. Recognition of dynamic and static facial expressions of emotion among older adults with major depression. Trends Psychiatry Psychother. (2019) 41:159–66. 10.1590/2237-6089-2018-0054 30942267

[22] Bourke C Douglas K Porter R. Processing of facial emotion expression in major depression: a review. Aust N Z J Psychiatry. (2010) 44:681–96. 10.3109/00048674.2010.496359 20636189

[23] Gollan JK Pane HT McCloskey MS Coccaro EF. Identifying differences in biased affective information processing in major depression. Psychiatry Res. (2008) 159:18–24. 10.1016/j.psychres.2007.06.011 18342954 PMC2571942

[24] Münkler P Rothkirch M Dalati Y Schmack K Sterzer P. Biased recognition of facial affect in patients with major depressive disorder reflects clinical state. PLoS ONE. (2015) 10:e0129863. 10.1371/journal.pone.0129863 26039710 PMC4454562

[25] Kiesler DJ The 1982 interpersonal circle: a taxonomy for complementarity in human transactions. Psychol Rev. (1983) 90:185–214. 10.1037/0033-295X.90.3.185

[26] Bird T Tarsia M Schwannauer M. Interpersonal styles in major and chronic depression: a systematic review and meta-analysis. J Affect Disord. (2018) 239:93–101. 10.1016/j.jad.2018.05.057 29990668

[27] Constantino MJ Manber R DeGeorge J McBride C Ravitz P Zuroff DC. Interpersonal styles of chronically depressed outpatients: profiles and therapeutic change. Psychother Theory Res Pract Train. (2008) 45:491–506. 10.1037/a0014335 22122536

[28] Sondermann S Stahl J Grave U Outzen J Moritz S Klein JP. Preoperational thinking as a measure of social cognition is associated with long-term course of depressive symptoms. A longitudinal study involving patients with depression and healthy controls. Front Psychiatry. (2020) 11:652. 10.3389/fpsyt.2020.00652 32733297 PMC7360820

[29] Liu RT. Childhood adversities and depression in adulthood: current findings and future directions. Clin Psychol Sci Pract. (2017) 24:140–53. 10.1111/cpsp.12190 28924333 PMC5600284

[30] Germine L Dunn EC McLaughlin KA Smoller JW. Childhood Adversity Is Associated with Adult Theory of Mind and Social Affiliation, but Not Face Processing. PLoS ONE. (2015) 10:e0129612. 10.1371/journal.pone.0129612 26068107 PMC4466913

[31] Petersen R Brakoulias V Langdon R. An experimental investigation of mentalization ability in borderline personality disorder. Compr Psychiatry. (2016) 64:12–21. 10.1016/j.comppsych.2015.10.004 26608042

[32] Rnic K Sabbagh MA Washburn D Bagby RM Ravindran A Kennedy JL. Childhood emotional abuse, physical abuse, and neglect are associated with theory of mind decoding accuracy in young adults with depression. Psychiatry Res. (2018) 268:501–7. 10.1016/j.psychres.2018.07.045 30165325

[33] Simon M Németh N Gálber M Lakner E Csernela E Tényi T. Childhood adversity impairs theory of mind abilities in adult patients with major depressive disorder. Front Psychiatry. (2019) 10:867. 10.3389/fpsyt.2019.00867 31920739 PMC6928114

[34] da Silva Ferreira GC Crippa JAS de Lima Osório F. Facial emotion processing and recognition among maltreated children: a systematic literature review. Front Psychol. (2014) 5:1–10. 10.3389/fpsyg.2014.01460 25566138 PMC4269127

[35] Gibb BE Schofield CA Coles ME. Reported history of childhood abuse and young adults' information processing biases for facial displays of emotion. Child Maltreat. (2009) 14:148–56. 10.1177/1077559508326358 18988860 PMC4077288

[36] Pollak SD Messner M Kistler DJ Cohn JF. Development of perceptual expertise in emotion recognition. Cognition. (2009) 110:242–7. 10.1016/j.cognition.2008.10.010 19059585 PMC2673797

[37] Pollak SD Tolley-Schell SA. Selective attention to facial emotion in physically abused children. J Abnorm Psychol. (2003) 112:323–38. 10.1037/0021-843X.112.3.323 12943012

[38] Rokita KI Dauvermann MR Donohoe G. Early life experiences and social cognition in major psychiatric disorders: a systematic review. Eur Psychiatry. (2018) 53:123–33. 10.1016/j.eurpsy.2018.06.006 30144982

[39] Christ C De Waal MM Dekker JJM van Kuijk I Van Schaik DJF Kikkert MJ. Linking childhood emotional abuse and depressive symptoms: the role of emotion dysregulation and interpersonal problems. PLoS ONE. (2019) 14:1–18. 10.1371/journal.pone.0211882 30763360 PMC6375578

[40] Huh HJ Kim SY Yu JJ Chae JH. Childhood trauma and adult interpersonal relationship problems in patients with depression and anxiety disorders. Ann Gen Psychiatry. (2014) 13:26. 10.1186/s12991-014-0026-y 25648979 PMC4304140

[41] Paradis A Boucher S Child maltreatment history and interpersonal problems in adult couple relationships. J Aggress Maltreat Trauma. (2010) 19:138–58. 10.1080/10926770903539433

[42] Klein JP Stahl J Hüppe M McCullough JP Schramm E Ortel D. Do interpersonal fears mediate the association between childhood maltreatment and interpersonal skills deficits? A matched cross-sectional analysis. Psychother Res. (2020) 30:267–78. 10.1080/10503307.2018.1532125 30309293

[43] Wittchen HU Wunderlich U Gruschwitz S Zaudig M SKID I. Strukturiertes Klinisches Interview für DSM-IV. Achse I: Psychische Störungen. Interviewheft und Beurteilungsheft. Eine deutschsprachige, erweiterte Bearb. d. amerikanischen Originalversion des SKID I. Göttingen: Hogrefe (1997).

[44] Klein JP Backenstrass M Schramm E Therapie-Tools CBASP. Basel: Beltz Verlag (2018).

[45] Beck AT Steer RA Brown GK Manual for the Beck Depression Inventory-II, Vol. 1 San Antonio: TX Psychol Corp (1996). p. 82 10.1037/t00742-000

[46] Hautzinger M Keller F Kühner C. Beck Depressions-Inventar (BDI-II). Frankfurt: Harcourt Test Services (2006).

[47] Kühner C Bürger C Keller F Hautzinger M. Reliabilität und validität des revidierten Beck- Depressionsinventars (BDI-II). Befunde aus deutschsprachigen stichproben Nervenarzt. (2007) 78:651–6. 10.1007/s00115-006-2098-7 16832698

[48] Bernstein DP Stein JA Newcomb MD Walker E Pogge D Ahluvalia T. Development and validation of a brief screening version of the childhood trauma questionnaire. Child Abus Negl. (2003) 27:169–90. 10.1016/S0145-2134(02)00541-0 12615092

[49] Wingenfeld K Spitzer C Mensebach C Grabe HJ Hill A Gast U The German version of the childhood trauma questionnaire (CTQ): preliminary psychometric properties. PPmP Psychother Psychosom Medizinische Psychol. (2010) 60:442–50. 10.1055/s-0030-1247564 20200804

[50] Davis MH A mulitdimensional approach to individual differences in empathy. J Pers Soc Psychol. (1980) 44:113–26.

[51] Paulus C (2009). Der Saarbrücker Persönlichkeitsfragebogen (SPF). Psychometrische Evaluation der deutschen Version des Interpersonal Reactivity Index. p. 1–11. Retrieved from: http://hdl.handle.net/20.500.11780/3343

[52] Baron-Cohen S Wheelwright S Hill J Raste Y Plumb I The reading the mind in the eyes test revised version: a study with normal adults, and adults with Asperger syndrome or high-functioning autism. J Child Psychol Psychiatry. (2001) 42:241–51. 10.1111/1469-7610.00715 11280420

[53] Kometer M Schmidt A Bachmann R Studerus E Seifritz E Vollenweider FX. Psilocybin biases facial recognition, goal-directed behavior, and mood state toward positive relative to negative emotions through different serotonergic subreceptors. Biol Psychiatry. (2012) 72:898–906. 10.1016/j.biopsych.2012.04.005 22578254

[54] Harmer CJ O'Sullivan U Favaron E Massey-Chase R Ayres R Reinecke A. Effect of acute antidepressant administration on negative affective bias in depressed patients. Am J Psychiatry. (2009) 166:1178–84. 10.1176/appi.ajp.2009.09020149 19755572

[55] Ekman P Friesen WV. Pictures of Facial Affect. Palo Alto, CA: Consulting Psychologist Press (1976).

[56] Horowitz LM Strauß B Thomas A Kordy H IIP-D Inventar zur Erfassung Interpersonaler Probleme-Deutsche Version [Inventory for the assessment of interpersonel problems-German version](Vol. 3, überarbeitete 3rd Auflage). Hogrefe: Boston, MA (2016).

[57] Thomas A Brähler E Strauß B IIP-32: Entwicklung, Validierung und Normierung einer Kurzform des Inventars zur Erfassung interpersonaler Probleme. Diagnostica. (2011) 57:68–83. 10.1026/0012-1924/a000034

[58] Hayes AF Introduction to Mediation, Moderation, and Conditional Process Analysis: A Regression-Based Approach. New York, NY: Guilford Publications (2017).

[59] Gambin M Sharp C. The relations between empathy, guilt, shame and depression in inpatient adolescents. J Affect Disord. (2018) 241:381–7. 10.1016/j.jad.2018.08.068 30145508

[60] Tone EB Tully EC. Empathy as a risky strength: a multilevel examination of empathy and risk for internalizing disorders. Dev Psychopathol. (2014) 26:1547–65. 10.1017/S0954579414001199 25422978 PMC4340688

[61] Powell PA. Individual differences in emotion regulation moderate the associations between empathy and affective distress. Motiv Emot. (2018) 42:602–13. 10.1007/s11031-018-9684-4 29899583 PMC5982456

[62] Banzhaf C Hoffmann F Kanske P Fan Y Walter H Spengler S. Interacting and dissociable effects of alexithymia and depression on empathy. Psychiatry Res. (2018) 270:631–8. 10.1016/j.psychres.2018.10.045 30384283

[63] Kettle JWL O'Brien-Simpson L Allen NB. Impaired theory of mind in first-episode schizophrenia: comparison with community, university and depressed controls. Schizophr Res. (2008) 99:96–102. 10.1016/j.schres.2007.11.011 18155447

[64] Nejati V Zabihzadeh A Maleki G Tehranchi A Mind reading and mindfulness deficits in patients with major depression disorder. Procedia Soc Behav Sci. (2012) 32:431–7. 10.1016/j.sbspro.2012.01.065

[65] Szily E Kéri S. Anomalous subjective experience and psychosis risk in young depressed patients. Psychopathology. (2009) 42:229–35. 10.1159/000218520 19451755

[66] Wolkenstein L Schönenberg M Schirm E Hautzinger M. I can see what you feel, but i can't deal with it: impaired theory of mind in depression. J Affect Disord. (2011) 132:104–11. 10.1016/j.jad.2011.02.010 21420177

[67] Oakley BFM Brewer R Bird G Catmur C. Theory of mind is not theory of emotion: a cautionary note on the reading the mind in the eyes test. J Abnorm Psychol. (2016) 125:818–23. 10.1037/abn0000182 27505409 PMC4976760

[68] McLaughlin KA Sheridan MA Lambert HK. Childhood adversity and neural development: deprivation and threat as distinct dimensions of early experience. Neurosci Biobehav Rev. (2014) 47:578–91. 10.1016/j.neubiorev.2014.10.012 25454359 PMC4308474

[69] Santini ZI Koyanagi A Tyrovolas S Mason C Haro JM. The association between social relationships and depression: a systematic review. J Affect Disord. (2015) 175:53–65. 10.1016/j.jad.2014.12.049 25594512

[70] Struck N Krug A Feldmann M Yuksel D Stein F Schmitt S. Attachment and social support mediate the association between childhood maltreatment and depressive symptoms. J Affect Disord. (2020) 273:310–7. 10.1016/j.jad.2020.04.041 32421618

[71] Wilbertz G Brakemeier EL Zobel I Härter M Schramm E. Exploring preoperational features in chronic depression. J Affect Disord. (2010) 124:262–9. 10.1016/j.jad.2009.11.021 20089311

[72] Rütgen M Pletti C Tik M Kraus C Pfabigan DM Sladky R. Antidepressant treatment, not depression, leads to reductions in behavioral and neural responses to pain empathy. Transl Psychiatry. (2019) 9:164. 10.1038/s41398-019-0496-4 31175273 PMC6555809

[73] Linehan MM Cognitive-Behavioral Treatment of Borderline Personality Disorder. New York, NY: Guilford Publications (2018).

