# Supplementary material for: Social Cognition and Interpersonal Problems in Persistent Depressive Disorder vs. Episodic Depression: The Role of Childhood Maltreatment
Source: Front Psychiatry. 2021 Jan 25;11:608795. doi: 10.3389/fpsyt.2020.608795 (PMC7873909; doi:10.3389/fpsyt.2020.608795)
Supplement: Supplementary file 1 [file Data_Sheet_1.docx]

**Supplementary Material**

**S1** **Table**

Comparison of social cognition and interpersonal problems between groups – additional subscales.

|  | |  | |  | | |  | | |  | | |  | | |  | |  | |
| --- | --- | --- | --- | --- | --- | --- | --- | --- | --- | --- | --- | --- | --- | --- | --- | --- | --- | --- | --- |
|  | | Group | | | | | | | | |  | | | | Effect size | | | | |
|  | | HC (*n* = 39) | | | ED (*n* = 38) | | | PDD (*n* = 34) | | | | Test statistic  *F_2,108_* | | HC vs ED  *d* | | | HC vs PDD  *d* | | ED vs PDD  d |
| Characteristic | | *M* | *SD* | | *M* | *SD* | | *M* | *SD* | | |  |  |  |  |  |  |  |  |
| Empathy (IRI) | |  |  | |  |  | |  |  | | |  | |  | | |  | |  |
| Fantasy | | 3.13 | 0.88 | | 3.06 | 0.79 | | 2.92 | 0.77 | | | 0.61 | | −0.08 | | | −0.25 | | −0.18 |
| Affective ToM (RMET) | | |  | |  |  | |  |  | | |  | |  | | |  | |  |
| Positive | | 2.85 | 1.65 | | 3.21 | 1.63 | | 2.71 | 1.45 | | | 0.99 | | 0.22 | | | −0.09 | | −0.32 |
| Negative | | 4.92 | 2.14 | | 4.24 | 1.98 | | 4.24 | 2.13 | | | 1.37 | | −0.33 | | | −0.32 | | 0 |
| Neutral | | 4.87 | 2.33 | | 5.39 | 1.99 | | 4.79 | 1.86 | | | 0.92 | | 0.24 | | | 0.04 | | −0.31 |
| Emotion Recognition Accuracy (FERT) ^a^ | | | | | | | |  |  | | |  | |  | | |  | |  |
| Disgust ^a^ | 51.49 | | 20.97 | | 50.43 | 16.16 | | 51.17 | 18.80 | | | 0.03 | | −0.06 | | | −0.02 | | 0.04 |
| Fear ^a^ | 37.16 | | 20.06 | | 41.21 | 12.52 | | 39.42 | 20.14 | | | 0.54^c^ | | 0.24 | | | 0.11 | | −0.11 |
| Surprise ^a^ | 64.73 | | 10.44 | | 63.64 | 10.90 | | 63.83 | 8.09 | | | 0.12 | | −0.10 | | | −0.10 | | 0.02 |
| Neutral ^a^ | 73.78 | | 16.05 | | 71.71 | 16.54 | | 66.33 | 22.97 | | | 1.40 | | −0.13 | | | −0.38 | | −0.27 |
| Interpersonal Problems (IIP) | | | | |  |  | |  |  | | |  | |  | | |  | |  |
| PA/ domineering | | 0.68 | 0.56 | | 1.23 | 0.77 | | 1.02 | 0.69 | | | 6.17** | | 0.82** | | | 0.54 | | −0.29 |
| BC/ vindictive | | 0.88 | 0.66 | | 1.12 | 0.65 | | 1.14 | 0.72 | | | 1.69 | | 0.37 | | | 0.38 | | 0.03 |
| LM/ self-sacrificing | | 1.56 | 0.87 | | 2.41 | 0.84 | | 2.68 | 0.77 | | | 17.95*** | | 0.99*** | | | 1.36*** | | 0.34 |
| NO/ intrusive | | 1.18 | 0.78 | | 1.97 | 0.74 | | 2.04 | 0.78 | | | 14.29*** | | 1.04***  *Note*s. CTQ = Childhood Trauma Questionnaire; HC = healthy control group; ED = episodic depression; PDD = persistent depressive disorder; IRI = Interpersonal Reactivity Index; FERT = Facial Expression Task; IIP = Inventory of Interpersonal Problems; ^a^ *N* = 102 (HC *n* = 37, ED *n* = 35, PDD *n* = 30), ^b^ *n* = 107, ^c^ Welch−ANOVA; Bonferroni Pos−hoc Tests for all comparisons; * p < .05, ** p < .01, *** p < .001 | | | 1.10*** | | 0.09 |


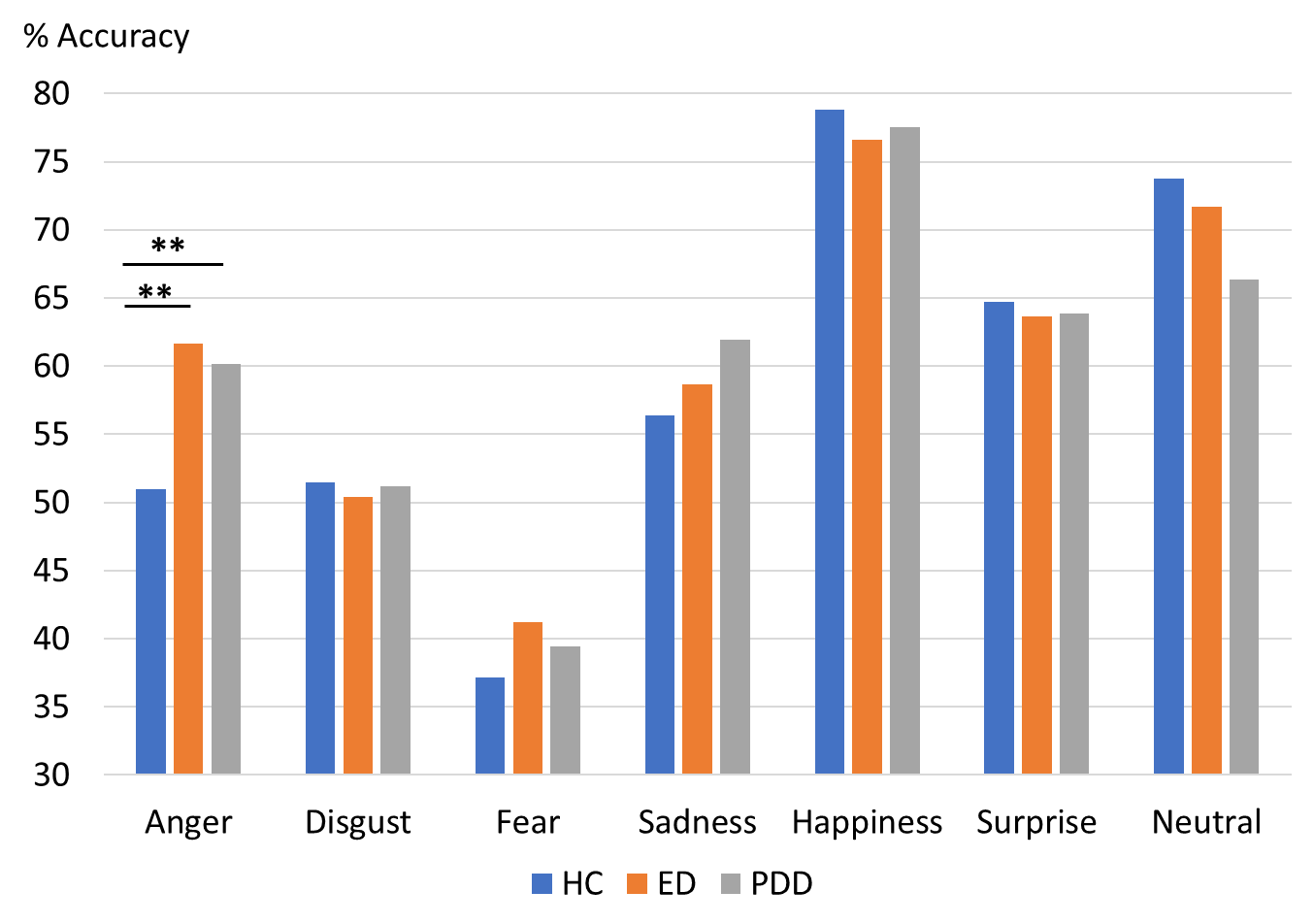

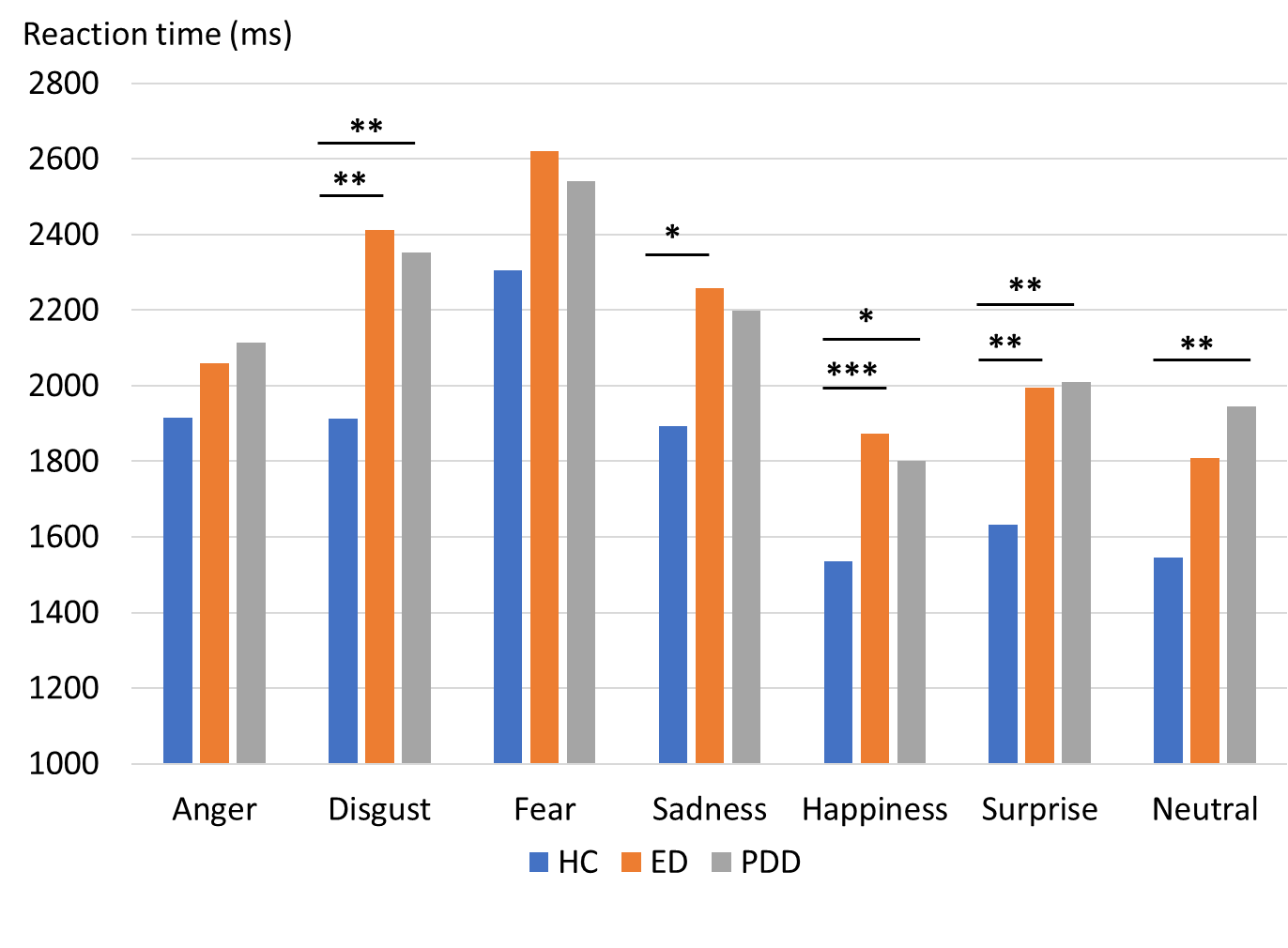


**S2 Figure.** Recognition accuracy (left) and reaction times (right) for the six facial expressions of emotion and neutral expressions in healthy controls (HC) and patients with episodic depression (ED) and persistent depressive disorder (PDD).

| **Table S3**  Bivariate correlations between childhood maltreatment, social cognitive variables, interpersonal problems, and depression. | | | | | | | | | | |
| --- | --- | --- | --- | --- | --- | --- | --- | --- | --- | --- |
| Variable | 1 | 2 | 3 | 4 | 5 | 6 | 7 | 8 | 9 | 10 |
|  |  |  |  |  |  |  |  |  |  |  |
| 1. Childhood   Maltreatment | 1 |  |  |  |  |  |  |  |  |  |
| 2. Empathic Concern | .25** | 1 |  |  |  |  |  |  |  |  |
| 3. Perspective Taking | −.20* | .23* | 1 |  |  |  |  |  |  |  |
| 4. Empathic Distress | .46*** | .28** | −.22* | 1 |  |  |  |  |  |  |
| 5. RMET errors | −.07 | −.12 | −.04 | −.05 | 1 |  |  |  |  |  |
| 6. Anger accuracy ^a^ | .08 | .12 | −.07 | .18 | −.24* | 1 |  |  |  |  |
| 7. Happiness accuracy ^a^ | −.19† | −.04 | .03 | −19† | −.26** | .19† | 1 |  |  |  |
| 8. Sadness accuracy ^a^ | .10 | .00 | .08 | .07 | −.31** | .35*** | .21* | 1 |  |  |
| 9. FERT global accuracy ^a^ | −.03 | .13 | .12 | .07 | −.43*** | .69*** | .41*** | .64*** | 1 |  |
| 10. Interpersonal   Problems ^b^ | .43*** | .22* | −.33** | .76*** | .03 | .11 | −.12 | .11 | <.01 | 1 |
| 11. Depression | .54*** | .38*** | −.20* | .74*** | −.09 | .25* | −.06 | .20* | .15 | .74*** |
| *Notes.* RMET = Reading the Mind in the Eyes Test; FERT = Facial Expression Recognition Task; † p < .06, * p < .05, ** p < .01, *** p < .001. | | | | | | | | | | |

| **Table S4** Partial correlations between childhood maltreatment and different facets of interpersonal problems, controlled for age and gender. | | | | | | | | | | | | | | | | | |
| --- | --- | --- | --- | --- | --- | --- | --- | --- | --- | --- | --- | --- | --- | --- | --- | --- | --- |
| Variable | 1 | | 2 | | 3 | | 4 | | 5 | | 6 | | 7 | | 8 | |  |
|  |  |  | |  | |  | |  | |  | |  | |  | |  | |
| 1. Childhood   Maltreatment | 1 | |  | |  | |  | |  | |  | |  | |  | |  |
| 2. Domineering/  controlling (PA) | .16 | | 1 | |  | |  | |  | |  | |  | |  | |  |
| 3. Vindictive/  self-centered (BC) | .09 | | .39*** | | 1 | |  | |  | |  | |  | |  | |  |
| 4. Cold/distant (DE) | .32** | | .31** | | .58*** | | 1 | |  | |  | |  | |  | |  |
| 5. Socially inhibited/  avoidant (FG) | .41*** | | .26** | | .47*** | | .75*** | | 1 | |  | |  | |  | |  |
| 6. Nonassertive (HI) | .28** | | .00 | | .20* | | .37*** | | .59*** | | 1 | |  | |  | |  |
| 7. Accomodating/  exploitable (JK) | .32** | | .04 | | .16 | | .34*** | | .59*** | | .77*** | | 1 | |  | |  |
| 8. Self-sacrificing/  nurturant (LM) | .35*** | | .23* | | .10 | | .24* | | .46*** | | .48*** | | .58*** | | 1 | |  |
| 9. Intrusive/ needy (NO) | .31** | | .44*** | | .25** | | .26** | | .33** | | .21* | | .33** | | .63*** | |  |
| *Notes.* * p < .05, ** p < .01, *** p < .001. | | | | | | | | | | | | | | | | | |
